# If the Test Fits: Diagnosis to Aid in the Treatment of Influenza

**DOI:** 10.7759/cureus.27850

**Published:** 2022-08-10

**Authors:** Raul Rodriguez, Diana Espinoza, Christine Junia

**Affiliations:** 1 Internal Medicine, MacNeal Hospital, Berwyn, USA

**Keywords:** influenza, ah1n1, pcr, ards

## Abstract

Influenza is a significant cause of morbidity and mortality worldwide, presenting with lethal complications such as acute respiratory distress syndrome (ARDS). Multiple ways to diagnose influenza include rapid antigen tests, flu polymerase chain reaction (PCR), and respiratory viral panels or multiplex PCR. However, they have different sensitivities and specificities. We present a case of a 70-year-old female who was admitted to the ICU for ARDS and had a high pretest probability of influenza. She had an initial rapid flu antigen test that was negative and a negative flu PCR. However, she tested positive for influenza A (H1N1) with a respiratory viral panel. We as physicians should take into consideration the different sensitivities and specificities diagnostic tests have and consider retesting patients who have a negative test in the context of a high pretest probability. We should also remember to begin antiviral therapy early in a patient with high suspicion of influenza with a severe clinical presentation despite not having a confirmed diagnosis.

## Introduction

Influenza is a significant cause of morbidity and mortality worldwide. According to the CDC, the US prevalence of influenza was estimated to be 36.9-42.4 million in 2018-2019 [1]. Mortality was reported in 59,500 cases, including deaths caused by acute respiratory distress syndrome (ARDS). Infectious Disease Society of America (IDSA) guidelines emphasize on proper clinical suspicion, early diagnosis, and antiviral therapy in critically ill patients since timely therapy has been proven to decrease mortality risk [2]. We present a case of ARDS complicating a delayed diagnosis of influenza A (H1N1) pneumonia.

## Case presentation

**Figure 1:**
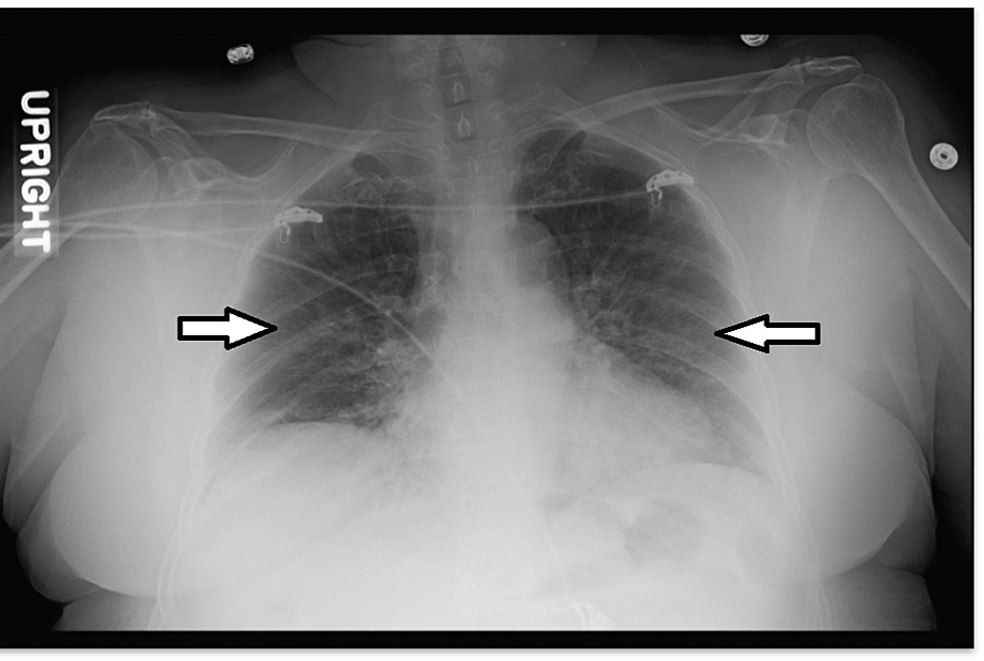
Chest X-ray on admission showing bilateral lung opacities and interstitial markings.

A 70-year-old female with no known medical history presented with productive cough, dyspnea, fever, and myalgia during the month of February. She had no known sick contacts, and her immunizations were not up-to-date. On physical exam, she had a temperature of 97.6 F, heart rate was 80 beats per minute (bpm), blood pressure (BP) was 113/76 mmHg, respiration rate (RR) was 18 bpm, and oxygen saturation (SatO2%) was 98%. No signs of respiratory distress with bilateral bibasilar crackles were observed. Complete blood count (CBC) and comprehensive metabolic panel (CMP) showed no significant lab abnormalities. Chest X-ray on admission revealed bilateral lung opacities and interstitial markings (Figure 1).

**Figure 2:**
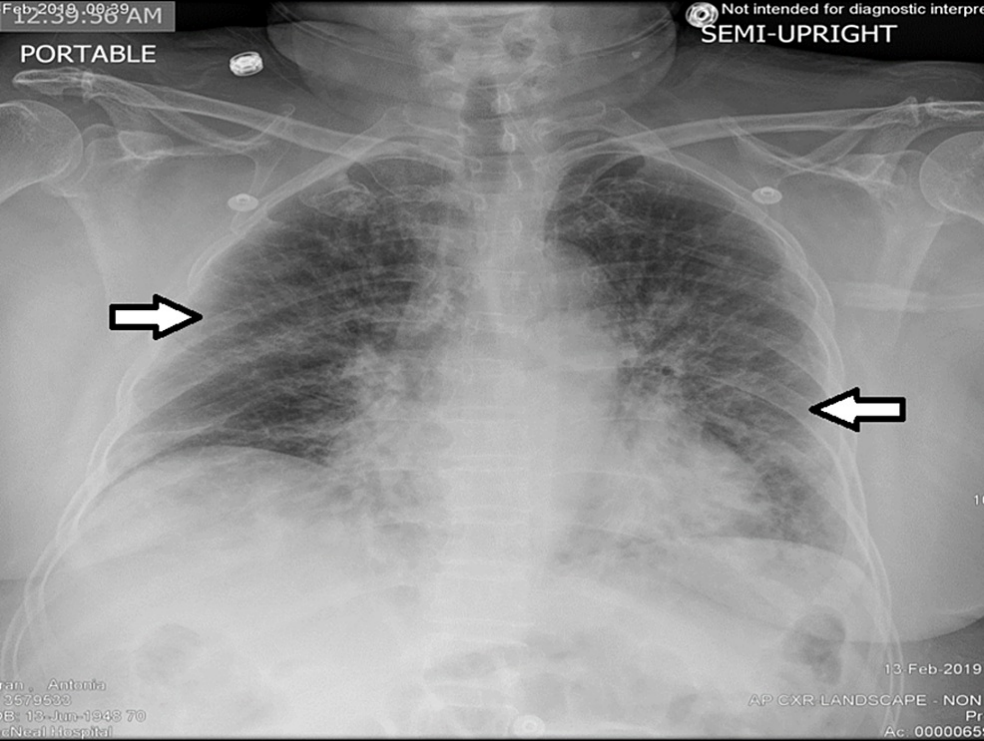
Chest X-ray on hospital day 3 showing diffuse bilateral infiltrates.

On admission, her differentials included community-acquired pneumonia (CAP) with viral vs. bacterial etiology. Initial testing included flu rapid antigen test and flu A/B/H1N1 PCR from nasal swab, both of which were negative. Therefore she was admitted for treatment of bacterial CAP and was started on ceftriaxone and azithromycin. However, by day 3 of hospitalization, she presented worsening shortness of breath and developed progressive hypoxia. A repeat chest X-ray was obtained (Figure 2), which showed diffuse bilateral infiltrates worsened compared to admission.

**Figure 3:**
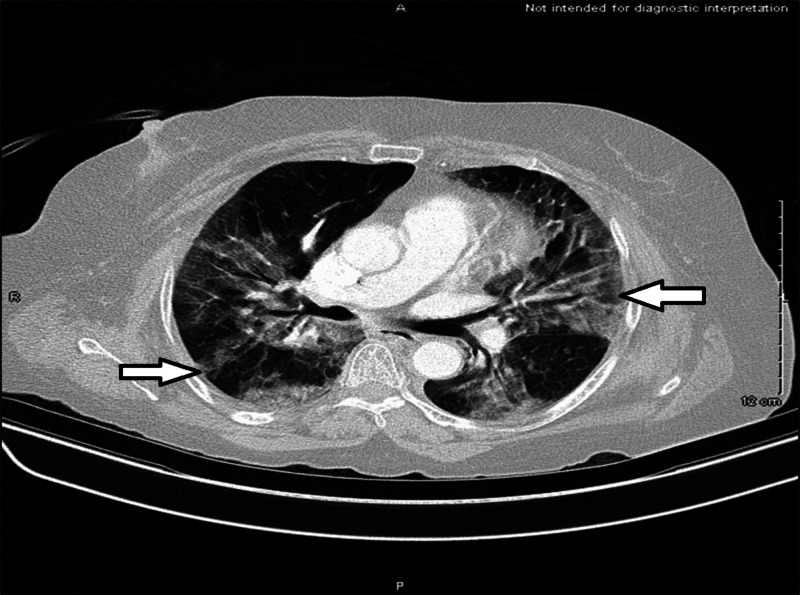
CT chest on hospital day 3 showing bilateral ground-glass opacities.

A CT chest was also obtained (Figure 3), which showed bilateral prominent ground-glass opacities.

An arterial blood gas (ABG) revealed a PaO2/FiO2 ratio of 149, consistent with moderate ARDS. The patient was then intubated and transferred to the ICU, she was put on low tidal volume ventilation, and her antibiotics were also broadened to vancomycin, aztreonam, and doxycycline for concern of sepsis secondary to bacterial CAP causing ARDS. Additional blood culture, sputum culture, and urinary antigens were ordered, which came back negative. Due to the persistence and worsening of her symptoms, a respiratory virus panel was sent on hospital day 4. The results came back positive for influenza A (H1N1), consistent with a diagnosis of influenza pneumonia complicated with ARDS. The patient was started on oseltamivir therapy by hospital day 5, but, unfortunately, continued to deteriorate clinically.

## Discussion

The CDC and IDSA have clear guidelines for diagnosing and managing influenza and its complications. Early diagnosis of influenza pneumonia is essential for initiating antiviral therapy to prevent life-threatening complications like ARDS [3]. For this purpose, multiple laboratory tests are available for diagnosis. RT-PCR has replaced viral cultures as the gold standard due to the rapid result availability and a greater sensitivity (of more than 90%) [4]. In comparison, rapid influenza diagnostic tests (RIDTs), also known as the rapid flu antigen test, have lower sensitivity (10-70%) and are therefore more suited for outpatient use. Multiplex RT-PCR assays also have high sensitivity and specificity (>90%) and the advantage of detecting influenza subtypes. Therefore, these tests are preferred in an inpatient setting. Their high sensitivity would imply that a negative PCR test would likely represent a true negative [4]. A meta-analysis of 162 studies that measured the sensitivity and specificity of multiple tests, including RIDT, direct immunoassay (DIA), and RT-PCR, showed that DIA and RT-PCR had a greater sensitivity of 80% and 91%, respectively, for the detection of influenza A, compared to 54.4% sensitivity for RIDTs [5].

The CDC and IDSA also recommend to start empiric antiviral therapy on patients hospitalized due to suspected or confirmed influenza [3-6]. Ideally, therapy would be started after the diagnosis of influenza is confirmed. However, it can be considered in the appropriate clinical context. This particularly applies to patients with a severe presentation (hypoxia, requiring intubation) and a high pretest probability. Side effects of neuraminidase inhibitors, particularly oseltamivir since it is the medication that is most commonly used, should also be considered. These include GI (nausea, vomiting), headaches, and hallucinations, though mostly in the younger population [7].

Despite numerous testing options available, our case highlights their limitation and the need for immediate medical management in cases where diagnosis must be made clinically. Our patient had a high pretest probability due to her symptom presentation, lack of response to appropriately expanded anti-bacterial therapy, and the seasonal epidemiology of influenza enough to make a clinical diagnosis [8]. Though PCR assays are highly sensitive, in a patient with a high pretest probability, false negatives may occur, in which case repeat or alternate testing might be warranted, be it with repeat flu PCR or respiratory viral panel. In our case, this led to an accurate diagnosis of influenza pneumonia complicated with ARDS.

## Conclusions

For patients with a high pretest probability for influenza with a suspected but not confirmed diagnosis with an unfavorable clinical presentation, we must consider starting empiric antiviral therapy to decrease mortality risk. It is also essential to repeat testing with the same or different assay in order to confirm the diagnosis in a patient with a negative result for influenza but has a high clinical suspicion. This can also be applied to other diseases in which a high pretest probability would preclude a negative test result despite high sensitivity.

## Appendices

**Table 1:** Basic metabolic panel on admission showing no abnormalities. Na: Sodium; Cl: Chloride; K: Potassium; HCO_3:_ Bicarbonate; Cr: Creatinine; BUN: Blood urea nitrogen.

Basic metabolic panel on admission	
Na	136 mmol/L (normal range: 135-145 mmol/L)
Cl	105 mmol/L (normal range: 95-110 mmol/L)
K	4.2 mmol/L (normal range: 3.5-5.5 mmol/L)
HCO_3_	24 mmol/L (normal range: 22-26 mmol/L)
Cr	1.06 mg/dl (normal range: 0.75-1.5 mmol/L)
BUN	20 mg/dl (normal range: 6-21 mmol/L)
Glucose	96 mg/dl (normal range: 70-100 mmol/L)
